# Mixed-Stable Models: An Application to High-Frequency Financial Data

**DOI:** 10.3390/e23060739

**Published:** 2021-06-11

**Authors:** Igoris Belovas, Leonidas Sakalauskas, Vadimas Starikovičius, Edward W. Sun

**Affiliations:** 1 Institute of Data Science and Digital Technologies, Faculty of Mathematics and Informatics, Vilnius University, LT-04812 Vilnius, Lithuania; 2 Department of Information Technologies, Faculty of Fundamental Sciences, Vilnius Gediminas Technical University, LT-2040 Vilnius, Lithuania; leonidas.sakalauskas@vilniustech.lt; 3 Department of Mathematical Modelling, Faculty of Fundamental Sciences, Vilnius Gediminas Technical University, LT-2040 Vilnius, Lithuania; vadimas.starikovicius@vilniustech.lt; 4 KEDGE Business School, Accounting, Finance, & Economics Department (CFE), Campus Bordeaux, 33405 Talence, France; edward.sun@kedgebs.com

**Keywords:** mixed-stable models, high-frequency data, stock index returns

## Abstract

The paper extends the study of applying the mixed-stable models to the analysis of large sets of high-frequency financial data. The empirical data under review are the German DAX stock index yearly log-returns series. Mixed-stable models for 29 DAX companies are constructed employing efficient parallel algorithms for the processing of long-term data series. The adequacy of the modeling is verified with the empirical characteristic function goodness-of-fit test. We propose the smart-Δ method for the calculation of the α-stable probability density function. We study the impact of the accuracy of the computation of the probability density function and the accuracy of ML-optimization on the results of the modeling and processing time. The obtained mixed-stable parameter estimates can be used for the construction of the optimal asset portfolio.

## 1. Introduction

The increased availability of high-frequency data has caused a great interest in the research of this subject. The main applications belong to financial engineering, ranging from risk management to options hedging, transaction execution, portfolio optimization, and forecasting. Thus, Bailey and Steeley [1] compared forecasts of the volatility of the Australian dollar exchange rate to alternative measures of ex post volatility, using high-frequency data. Degiannakis and Filis [2] examined the importance of combining high-frequency financial information, along with the oil market fundamentals, to gain incremental forecasting accuracy for oil prices, showing that although the oil market fundamentals are helpful for long-run forecasting horizons, the combination of the latter with high-frequency financial data significantly improve oil price forecasts. Zhang and Wang [3] employed the MIDAS model and the high-frequency data of four stock market indices to forecast WTI and Brent crude oil prices at a lower frequency. The results indicated that high-frequency stock market indices have a certain advantage over the lower-frequency data in forecasting monthly crude oil prices, and the MIDAS model using high-frequency data proves superior to the ordinary model.

Göncü and Yang compared variance-gamma and normal-inverse Gaussian distributions with the benchmark of generalized hyperbolic distribution in terms of their fit to the empirical distribution of Chinese high-frequency stock market index returns, showing that as the time scale of log-returns decrease, a normal-inverse Gaussian model consistently outperforms the variance-gamma model [4]. Remarkably, this result for the normal-inverse Gaussian model is consistent with findings of Belovas from the the same year [5], based on five Standard & Poor’s stock market indices (covering the period of 10 years, 2006–2016) log-returns. Koopman et al. [6] investigated how dependence between high-frequency price changes of financial stocks (10-second frequency for 10 US financial stocks for January 2012 to Decemeber 2012) varies within the day. Schabek et al. [7] examined high-frequency log-returns of the Zagreb Stock Exchange CROBEX Index (from September 2017 to March 2018) to assess the reactions of the index to macroeconomic announcements regarding the Croatian economy within an ultrashort time periods. Tony Cai et al. [8] examined the estimation of a high-dimensional minimum-variance portfolio based on the high-frequency returns from S&P 100 Index constituents during the years 2003–2013. Huang and Gao [9] used high-frequency Bitcoin trading data (from 1 January 2012 to 12 August 2019) to explore the Bitcoin return predictability.

Ongoing COVID-19 turmoil has set a new trend in high-frequency studies. Thus, Ambros et al. [10], using 30 min tick returns, studied the impact of changes in the number of COVID-19 news on eight different stock markets during the initial two months of the coronavirus crisis, showing that COVID-19-related news impact stock market volatility positively in Europe, but less so in other markets. Yousaf and Ali [11] analyzed return and volatility transmission between major cryptocurrencies (Bitcoin, Ethereum and Litecoin) during the pre-COVID-19 and COVID-19 periods (it is noteworthy that the authors advised the investors to decrease their investments in Bitcoin).

Methodologies based on high-frequency data analysis also can be found in neural science and real-time network traffic management (cf. Kaklauskas [12]). A summary of the literature covering high-frequency and intra-daily data research is presented in [13] and the references therein.

The paper extends the study of applying the mixed-stable models to the analysis of large sets of high-frequency financial data; see [4,14,15]. In this research, we apply the parallel computing approach (cf. [16]) to the mixed-stable modeling of high-frequency data. We often observe many zero returns in the high-frequency return data in practice because the underlying asset price does not change at given short-time intervals. The mixed-stable model is well suited to cope with this specific feature.

We introduce the smart-Δ approach to the calculation of the α-stable probability density function and consider the impact of the accuracy of the computation of the probability density function and the accuracy of the maximum-likelihood optimization on the results of the modeling and processing time.

The mixed-stable model for financial data was first applied in [17]. The preliminary research [18] was dedicated to the analysis of empirical data covering one specific day. In the present study, we analyze high-frequency data for the whole year. This will drastically increase the size of data sets and computing time. We address this issue using more efficient numerical methods and parallel algorithms.

The paper is organized as follows. The first part is the introduction. Section 2 describes the real data used in our research, their aggregation and their transforms. Section 3 introduces our modeling methodology and smart-Δ method for calculating the α-stable probability distribution function. In Section 4, we discuss the impact of computations’ and optimization’ accuracy on the modeling and processing time and present the modeling results. The last section is devoted to the concluding remarks.

## 2. Data

In the previous research of intra-daily data from German DAX component stock returns ([18]), we analyzed high-frequency data series of 29 stocks from DAX that represent just one business-active day of the year (17 August 2007). The present paper extends the research and deals with the whole year’s intra-daily data (from 1 January 2007 to 27 December 2007; 251 days in total). The year 2007 is of particular interest to economists and financial analysts. Moreover, empirical data of this year comprise a valuable test case in creating and testing special models, because 2007 is the first year of the Global Financial Crisis of 2007–2008. Before the current COVID-19 turmoil, it was considered by many experts to have been the most severe financial crisis since the Great Depression, contributing significantly to the Eurozone crisis.

We aggregate raw inhomogeneous intra-daily data into equally-spaced homogeneous intra-daily time series. The aggregation is done with the previous-tick interpolation. A linear interpolation relies on future information, whereas the previous-tick interpolation is based on the information already known (cf. [19]).

### 2.1. Previous-Tick Interpolation

We denote times of raw inhomogeneous intra-daily series as $\left\{t_{i}\right\}$ and the corresponding prices as $\left\{P_{i}\right\}$, where $P_{i}=P\left(t_{i}\right)$. The aggregated homogeneous high-frequency series $\left\{{\tilde{P}}_{j}\right\}$ is obtained at times ${\tilde{t}}_{j}=t_{0}+j\Delta t$ with the step Δt, where the index *j* identifies the regularly spaced sequence. By means of the previous-tick interpolation, we obtain that

(1) $${\tilde{P}}_{j}=P\left(\max\left\{t_{i}:\;t_{i}\leq {\tilde{t}}_{j}\right\}\right).$$

Having obtained equally-spaced price series, we can calculate the corresponding logarithmic returns series $\left\{X_{j}\right\}$:

$$X_{j}=\log\frac{{\tilde{P}}_{j}}{{\tilde{P}}_{j-1}}.$$

### 2.2. Models for Financial Data

Classical techniques in financial engineering heavily relied on the assumption that the random variables under investigation follow a normal distribution. However, time series observed in finance often deviate from the Gaussian model, exhibiting fat tails and asymmetry (cf. [18,19]). In such a situation, the classical approach’s appropriateness for the modeling of returns is highly questionable. On the other hand, financial asset returns are the cumulative outcome of a vast number of pieces of information and individual decisions arriving almost continuously. Hence, in the presence of heavy tails, it is natural to assume that they are approximately governed by a non-Gaussian distribution (cf. [20]).

We can distinguish several fundamental reasons why models with the α-stable paradigm are used in financial engineering. The first is that stable random variables justify the generalized central limit theorem, which states that stable distributions are the only asymptotic distributions for adequately scaled and centered sums of independent identically distributed random variables (see [20]). The second one is that stable distributions are heavy-tailed (cf. [18,19]). All but one of the stable distributions have infinite variance, which implies that observations of a large magnitude can be expected and may dominate the sums of these random variables. It is not correct to treat these observations as outliers, since their exclusion takes away much of the significance of the original data; indeed, it is specifically these observations that may be of the most significant interest. The third reason is that stable distributions are asymmetric and leptokurtic [21]. Since stable distributions can accommodate the heavy tails and asymmetry, they ensure a perfect fit for empirical data. They are particularly valuable models for data sets covering extreme events such as market crashes or natural catastrophes. The fourth reason is that stable distributions are a more flexible tool compared to the normal distribution. As was pointed out by Cont, a parametric model to successfully reproduce specific empirical features of asset returns must have at least four parameters: a parameter describing the decay of the tails (stability index), an asymmetry parameter allowing the left and right tails to behave differently, a scale (volatility) parameter and ultimately a location parameter [22]. Our recent research on the comparison of models corroborates this opinion [15].

### 2.3. Empirical Moments

Having processed yearly high-frequency returns data for 29 stocks at different time steps, we found that almost all data series are asymmetric (some typical examples may be found in Table 1), and the empirical kurtosis shows that density functions of the series are more peaked than Gaussian density functions.

However, it should be pointed out that rather often, empirical data exhibit the stagnation effect; i.e., series contain numerous zero returns. This phenomenon is especially characteristic of young emerging markets with low-liquidity financial instruments [17] and high-frequency financial data [18]. We have examined the obtained returns series for the stagnation effect. In Table 2, we show *max* and *min* lengths of returns series with zeros removed as well as *max* and *min* percent of zeros depending on the level of aggregation.

As we can see in Table 2, a strong stagnation effect (43 to 82 percent zeros at 10 s time step) manifests itself in most high-frequency series. To take the zero effect into account, we apply a generalized mixed-stable model.

## 3. Stable and Mixed-Stable Models

### 3.1. α-Stable Distribution

The α-stable distribution is usually described by its characteristic function φ(t):

(2) $$\log\varphi \left(t\right)=\left\{\begin{array}{cc} -\sigma^{\alpha }{\left|t\right|}^{\alpha }\left\{1-i\beta sign\left(t\right)\tan\frac{\pi \alpha }{2}\right\}+i\mu t, & \alpha \neq 1,\\ -\sigma |t|\{1+i\beta sign\left(t\right)\frac{2}{\pi }\log|t|\}+i\mu t, & \alpha =1, \end{array}\right.$$

where α∈(0,2], β∈[−1,1], σ>0, μ∈R. Here, α is the stability index (in financial modeling, it is generally assumed that 1<α≤2), β is the skewness, μ is the location parameter and σ is the scale parameter. If σ=1 and μ=0, then the distribution is called standard stable. An overview of stable distributions properties can be found in [20]. The probability density function of stable laws cannot be expressed in elementary functions, except for a few cases: Levy, Cauchy and Gaussian distributions.

The canonical representation (2) has one serious disadvantage. Characteristic functions have discontinuities at all points with α=1,β≠0. Therefore, for numerical purposes, it is advisable to use Nolan’s parametrization:

(3) $$\log\varphi_{0}\left(t\right)\;\;=\;\;\left\{\begin{array}{cc} -\sigma^{\alpha }{\left|t\right|}^{\alpha }(1\;+\;i\beta sign\left(t\right)\tan\frac{\pi \alpha }{2}{\left((\sigma |t|\right)}^{1-\alpha }-1))\;+\;i\mu_{0}t, & \alpha \neq 1,\\ \sigma |t|(1+i\beta sign\left(t\right)\frac{2}{\pi }\log(\sigma |t|))+i\mu_{0}t, & \alpha =1. \end{array}\right.$$

This parametrization is a variant of Zolotarev’s (M) parametrization, with the density and the distribution function jointly continuous in all the four parameters ([20]). The location parameters of these two representations are related by

$$\mu_{0}=\left\{\begin{array}{cc} \mu +\beta \sigma \tan\frac{\pi \alpha }{2}, & \alpha \neq 1,\\ \mu +\beta \sigma \frac{2}{\pi }\ln\sigma , & \alpha =1. \end{array}\right.$$

The probability distribution function of representations (2) and (3) are related by

(4) $$p\left(x,\Theta \right)=\frac{1}{\sigma }p_{0}\left(\frac{x-\mu_{0}}{\sigma },\alpha ,\beta \right).$$

Here, Θ=(α,β,μ,σ), and $p_{0}\left(x,\alpha ,\beta \right)$ is Nolan’s standard stable probability distribution function with the integral representation

(5) $$p_{0}\left(x,\alpha ,\beta \right)=\frac{1}{\pi }\int_{0}^{\infty }\exp\left(-t^{\alpha }\right)\cos\left(h\left(x,t;\alpha ,\beta \right)\right)dt,$$

where

$$h\left(x,t;\alpha ,\beta \right)=\left\{\begin{array}{cc} xt+\beta \tan\frac{\pi \alpha }{2}\left(t-t^{\alpha }\right), & \alpha \neq 1,\\ xt-\beta t\frac{2}{\pi }\ln t, & \alpha =1. \end{array}\right.$$

A precise and fast calculation of stable densities is a nontrivial task (cf. [16,20]). To deal with the integral representation of the probability density function of α-stable distribution (5), we introduce the smart-Δ approach, replacing the improper integral in (5) by a definite integral with the upper integration bound Δ=Δ(α,ε). Here, α is the stability index, and by ε we denote the error of the approximation. Details of the technique are explained in Section 3.2.

The problem of parameter estimation in stable modeling is hampered by the lack of closed form for stable density functions. Hence, many statistical methods depending on the probability density function’s explicit form cannot be applied. Comparative studies (see [23]) corroborate that the most accurate method of estimation is the maximum likelihood method. However, it is the most time-consuming. The vector of stable parameters Θ=(α,β,μ,σ) can be estimated from the returns $\left\{X_{j}\right\}$ by maximizing the log-likelihood function

(6) $$L\left(\Theta \right)=\sum_{k=1}^{n}\ln p\left(X_{k},\Theta \right).$$

In [16], we have studied the log-likelihood target function profiles for artificially generated stable distributed data. We have obtained that the log-likelihood target function is of an uniextremal nature, often with a very flat surface in the extremum’s neighborhood. In the present research, we have examined the log-likelihood target function for real financial data. Target function (6) was calculated for returns series Allianz SE of size 7385, which was obtained using the previous-tick interpolation (1) with the time step Δt=1000. The ML solution vector for this data set is

α=1.541470,β=0.004055,μ=0.000005,σ=0.001241.

**Figure 1:**
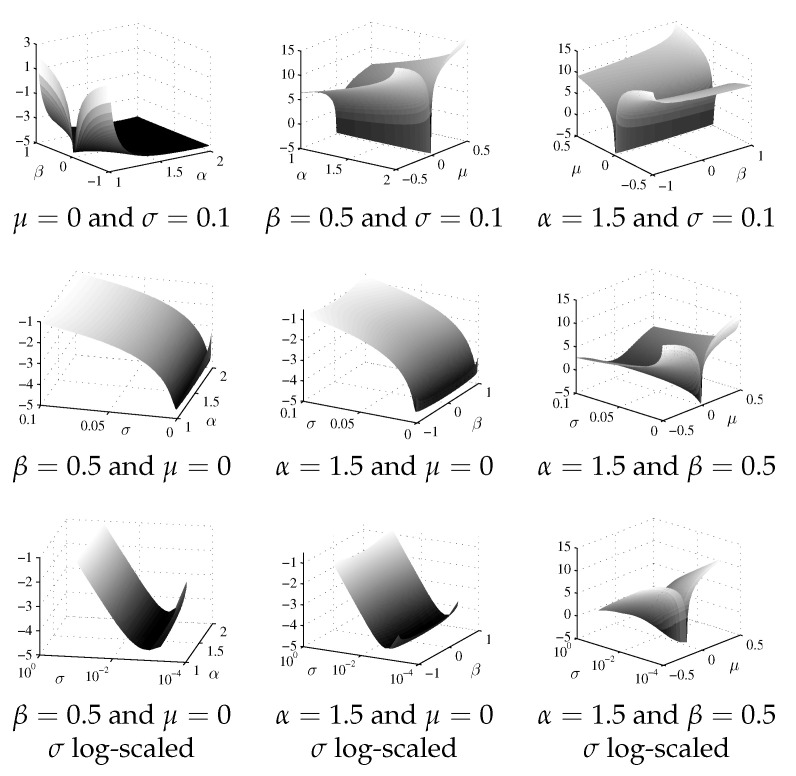
3D cuts of the log-likelihood target function (6), obtained by fixing pairs of parameters.

Figure 1 shows 3D cuts of the target function, obtained by fixing pairs of parameters. As one can see, the target function with real data exhibits qualitatively the same behavior as with the artificially generated data (cf. [16]).

To optimize the log-likelihood function, we use the Nelder-Mead method. Although this method is not the fastest one, it is robust and does not require the calculation of derivatives (gradient or Hessian).

### 3.2. Stable Probability Density Function Calculation: The Smart-Δ Approach

The lack of analytical representation of the probability distribution function (with a few exceptions: Gaussian, Cauchy and Levy distributions) hampers the practical implementation of stable models. We can evaluate the stable probability density function replacing the improper integral (5)

$$p_{0}\left(x,\alpha ,\beta \right)=\frac{1}{\pi }\int_{0}^{\infty }\exp\left(-t^{\alpha }\right)\cos\left(h\left(x,t;\alpha ,\beta \right)\right)dt$$

with an approximation $I_{0}$,

(7) $$I_{0}=\frac{1}{\pi }\int_{0}^{\Delta }\exp\left(-t^{\alpha }\right)\cos\left(h\left(x,t;\alpha ,\beta \right)\right)dt,$$

with a tail error

(8) $$I_{\Delta }=\frac{1}{\pi }\int_{\Delta }^{\infty }\exp\left(-t^{\alpha }\right)\cos\left(h\left(x,t;\alpha ,\beta \right)\right)dt.$$

Calculating $I_{0}$ with $\frac{\varepsilon }{2}$ precision and dropping $I_{\Delta }$ evaluated with the same accuracy yields ε joint accuracy for the probability density function. For α>1, as is usually assumed in financial engineering, we have

$$|I_{\Delta }|⩽\frac{1}{\pi }\int_{\Delta }^{\infty }\exp\left(-t^{\alpha }\right)dt⩽\frac{1}{\pi }\int_{\Delta }^{\infty }\exp\left(-t\right)dt=\frac{1}{\pi }\exp\left(-\Delta \right).$$

Hence, the roughest way of evaluating Δ (see [16]) is

$$\Delta \approx -\ln\frac{\pi \varepsilon }{2}.$$

The error of the approximation does not exceed ε/2. Noticing a relation of the bound of $I_{\Delta }$ to an upper incomplete gamma function Γ(s,x),

$$|I_{\Delta }|⩽\frac{1}{\pi }\int_{\Delta }^{\infty }\exp\left(-t^{\alpha }\right)dt=\frac{1}{\pi \alpha }\int_{\Delta^{\alpha }}^{\infty }u^{\frac{1}{\alpha }-1}e^{-u}du,$$

we can evaluate Δ=Δ(α,ε) in a more subtle way, as a root of a nonlinear equation

$$\Gamma \left(\frac{1}{\alpha },\Delta^{\alpha }\right)=\frac{\alpha \pi \varepsilon }{2}.$$

We can calculate Δ as follows. For x→∞, we have $\Gamma \left(a,x\right)∼x^{a-1}e^{-x}$, yielding an approximate equation

$$\Delta^{\alpha \left(\frac{1}{\alpha }-1\right)}e^{-\Delta^{\alpha }}=\frac{\alpha \pi \varepsilon }{2},$$

or, unless α=1,

$$\frac{\alpha }{\alpha -1}\Delta^{\alpha }e^{\frac{\alpha }{\alpha -1}\Delta^{\alpha }}=\frac{\alpha }{\alpha -1}{\left(\frac{\alpha \pi \varepsilon }{2}\right)}^{\frac{\alpha }{1-\alpha }}.$$

Next, Δ can be expressed in terms of the Lambert *W* function, i.e.,

(9) $$\Delta =\Delta \left(\alpha ,\varepsilon \right)=\left\{\begin{array}{cc} -\ln\frac{\pi \varepsilon }{2}, & \alpha =1,\\ {\left(\frac{\alpha -1}{\alpha }W\left(\frac{\alpha }{\alpha -1}{\left(\frac{\alpha \pi \varepsilon }{2}\right)}^{\frac{\alpha }{1-\alpha }}\right)\right)}^{\frac{1}{\alpha }}, & \alpha \neq 1. \end{array}\right.$$

Here, W(x) stands for the Lambert *W* function ($y=W\left(x\right)\;\Leftrightarrow \;x=ye^{y}$). For x≥0, we can calculate the principal branch of the Lambert *W* function using Halley’s method (cf. Corless et al. [24]). If x<0 (i.e., α<1), then we apply an algorithm, proposed by [25], to calculate the branch $W_{-1}$. Note that in order to proceed from a standard stable density to a stable density, we interchange in expression (9) π coefficients with πσ.

### 3.3. Mixed-Stable Distribution

The mixed-stable model was introduced to deal with the problem of daily zero returns ([17]). The probability density function of a mixed-stable random variable is

(10) f(x,Θ)=(1−r)p(x,Θ)+rδ(x),

where p(x,Θ) is the probability density function (4) of a stable distribution (2) and δ(x) is the Dirac delta function. The coefficient r∈(0,1) is the index of stagnation.

The empirical cumulative distribution functions of data series with the stagnation effect exhibit jumps at x=0. Model (10) enables us to accommodate these jumps. The likelihood function of the mixed-stable model (10) is given by

$$l\left(r,\Theta \right)=C_{n}^{k}{\left(1-r\right)}^{n-k}r^{k}\prod_{j=1}^{n-k}p\left(x_{j},\Theta \right),$$

where $\left\{x_{1},x_{2},\ldots ,x_{n-k}\right\}$ is a non-zero returns set, obtained by excluding *k* zero returns from the initial returns set $\left\{X_{1},X_{2},\ldots ,X_{n}\right\}$. By optimizing the first factor ${\left(1-r\right)}^{n-k}r^{k}$, we obtain $r_{max}=k/n$. The optimization of the product is equivalent to the optimization of the likelihood function of the stable distribution of the non-zero returns set $\left\{x_{1},x_{2},\ldots ,x_{n-k}\right\}$. Hence the optimal vector $\Theta_{max}$ is estimated with non-zero returns via stable log-likelihood function (6).

Having parameters of the mixed-stable law estimated, we proceed with modeling adequacy testing. Since we have a discontinuous distribution function, classic methods for continuous distributions (e.g., Kolmogorov–Smirnov or Anderson–Darling tests) are unsuitable. Therefore, we apply a special goodness-of-fit test based on characteristic functions (note that characteristic functions are uniformly continuous on the entire space) proposed in [26].

## 4. Results and Discussion

Using the maximum-likelihood method (see Section 3), we have estimated parameters of mixed-stable models (10) for DAX financial data. Next, we studied the impact of the accuracy of the probability density function (4) calculation ($\varepsilon_{pdf}$) and the maximum-likelihood optimization ($\varepsilon_{ML}$) on the results of the modeling and processing time. We found that insufficient accuracy results in faulty outcomes. We illustrate the facts in Table 3, which contains Θ=(α,β,μ,σ) estimates for the Deutsche Post AG data series, taken with time step Δt=10 s. For every set of estimates, the corresponding processing time (PT) and the outcome of the Koutrouvelis goodness-of-fit test ([26]) for the adequacy of the modeling (KT) are indicated (the significance level of the test is 5%). As we see, we need at most $\varepsilon_{pdf}=10^{-9}$ and $\varepsilon_{ML}=10^{-6}$ to achieve plausible results. Further increase in accuracy levels shows the convergence of obtained estimates. For higher accuracies, at least seven significant digits are not changing.

Note that because of the flat surface of the ML-target function (see Figure 1), the initial guess selection is essential for fast and correct optimization. Thus, the global optimization (either deterministic or stochastic) is the natural way of solving this problem. However, it requires further in-depth investigation.

An increase in accuracies $\varepsilon_{pdf}$ and $\varepsilon_{ML}$ causes a surge in processing time (see Table 3). In Table 4, we show the optimization time for 29 DAX high-frequency returns series taken with the time step Δt=10 s. These results were obtained using parallel algorithms introduced with early-stage research ([14]) with 64 processes. Note that when $\varepsilon_{pdf}∼\varepsilon_{ML}$, convergence is naturally very bad. Next, $\varepsilon_{pdf}$ and $\varepsilon_{ML}$ cannot be chosen independently, We must select $\varepsilon_{pdf}$ in relation to $\varepsilon_{ML}$ (e.g., $\varepsilon_{pdf}=10^{-12}$ is sufficient for $\varepsilon_{ML}=10^{-7}$; however, the convergence stagnates if we choose $\varepsilon_{pdf}=10^{-12}$ for $\varepsilon_{ML}=10^{-8}$). The number of stagnated series for every accuracy pair ($\varepsilon_{pdf}$ and $\varepsilon_{ML}$) is given in parentheses next to the corresponding overall processing time (see Table 4).

Finally, we present the mixed-stable modeling results for 29 DAX companies. These results were obtained with $\varepsilon_{ML}=10^{-7}$ accuracy of the optimization method and $\varepsilon_{pdf}=10^{-12}$ accuracy of mixed-stable density function calculation. We must stress that we could obtain these results in a reasonable time using only parallel computations. Table 5 contains estimates of a stagnation parameter *r*, stable parameters Θ=(α,β,μ,σ) and the outcome of the Koutrouvelis test (zero stands for “rejected”, unity stands for “not rejected”).

The mixed-stable model, with a corresponding set of estimated parameters, was accepted for almost all DAX companies, justifying the application of mixed-stable models in high-frequency finance analysis (see Table 5). Note that considerable differences in corresponding parameter sets are obtained from yearly (cf. Table 5) and daily (cf. [18]) empirical data. This indicates the necessity of large data sets processing in forecasting and portfolio construction.

Examples of the use of the estimated mixed-stable parameters for the selection of the optimal asset portfolio have been provided in our preliminary comparative research [15]. This study compared stable and mixed-stable models with mixed diffusion-jump, the mixture of normals, scaled-*t*, logistic and normal-inverse Gaussian models and identified the mixed-stable one as the most adequate model for the data under analysis. The modeling results can be applied for the optimal portfolio selection employing two different strategies (considered in the research) with and without the relation coefficients matrices.

## 5. Conclusions

Having processed yearly high-frequency returns data for 29 German DAX companies with different time steps, we found that almost all data series are asymmetric. Moreover, the empirical kurtosis shows that density functions of the series are more peaked than Gaussian. We have noticed a stagnation effect in obtained high-frequency returns series. These factors lead us to the application of mixed-stable models. We introduced the smart-Δ upper integration bound to deal with the computationally demanding α-stable probability density function in ML-optimization. Parallel algorithms were employed to deal with sizeable yearly data sets.

We have studied the impact of pdf-computation accuracy and the accuracy of ML-optimization on the results of the modeling and processing time. We constructed mixed-stable models for all 29 DAX companies. The adequacy of models was tested with Koutrouvelis goodness-of-fit test based on the empirical characteristic functions. Almost all models were accepted with corresponding sets of estimated parameters, justifying the mixed-stable modeling in high-frequency data analysis. Obtained parameter estimates can be used in the construction of the optimal asset portfolio.

The next research objective is to compare the presented results (concerning the first year of the Global Financial Crisis of 2007–2008) with the planned analysis of ongoing COVID-19 crisis data. Two more points to be brought to the attention are the robustness of empirical results for different periods and comparative studies with other markets. 

## Figures and Tables

**Table 1:** Empirical moments for six DAX returns series with different time step Δt.

Company	Δt, Sec.	Mean	St. Dev.	Skewness	Kurtosis	Zeros, %
Adidas AG	10	$3.5949\times 10^{-7}$	0.0004	2.1461	704.98	75.32
	100	$3.6122\times 10^{-6}$	0.0011	2.3129	142.39	22.96
	1000	$3.6727\times 10^{-5}$	0.0029	0.5036	20.285	6.00
Deutsche Bank	10	$-1.7318\times 10^{-7}$	0.0004	−2.1778	1246.5	47.07
	100	$-1.7408\times 10^{-6}$	0.0010	−1.1853	253.86	9.15
	1000	$-1.8183\times 10^{-5}$	0.0031	−0.0737	31.182	2.59
BASF SE	10	$4.0699\times 10^{-7}$	0.0003	−2.3481	590.28	57.90
	100	$4.0647\times 10^{-6}$	0.0009	−0.6817	96.550	10.90
	1000	$4.1485\times 10^{-5}$	0.0027	0.4249	29.408	2.80
BMW AG St	10	$-4.5688\times 10^{-8}$	0.0004	−1.1297	870.09	67.96
	100	$-3.8533\times 10^{-7}$	0.0010	−0.9190	147.39	18.38
	1000	$-4.1137\times 10^{-6}$	0.0029	−0.2380	21.901	5.02
Deutsche Börse	10	$-6.0764\times 10^{-8}$	0.0009	−560.34	422,496	68.42
	100	$-6.2438\times 10^{-7}$	0.0028	−196.46	48,486.4	14.64
	1000	$-8.0474\times 10^{-6}$	0.0086	−64.547	5074.6	2.47
SAP AG	10	$-1.7207\times 10^{-7}$	0.0004	−13.423	2818.2	57.62
	100	$-1.7304\times 10^{-6}$	0.0011	−6.5862	566.56	17.93
	1000	$-1.7301\times 10^{-5}$	0.0031	−4.9374	170.55	6.14

**Table 2:** Lengths and stagnation (percent of zero returns) in yearly (1 January–27 December 2007) stock returns series with different time step Δt.

Δt, Sec.	*Min* Length	*Max* Length	*Min* Zero %	*Max* Zero %
10	135,001	436,143	43	82
100	3612	71,388	7	43
1000	6051	7385	2	20

**Table 3:** Dependence of mixed-stable estimates on the accuracy of the probability density function (4) calculation ($\varepsilon_{pdf}$) and the accuracy the maximum likelihood optimization ($\varepsilon_{ML}$); Deutsche Post AG returns data series with time step Δt=10 sec. Processing time (PT) (sec) and Koutrovelis test outcome (KT, 5% significance level).

		$\varepsilon_{\mathrm{pdf}}$				
$\varepsilon_{\mathrm{ML}}$		$10^{-8}$	$10^{-9}$	$10^{-10}$	$10^{-11}$	$10^{-12}$
$10^{-5}$	α	1.965528	1.965528	1.965528	1.965528	1.965528
	β	0.984687	0.984687	0.984687	0.984687	0.984687
	μ	0.000008	0.000008	0.000008	0.000008	0.000008
	σ	0.000531	0.000531	0.000531	0.000531	0.000531
	PT	310.16	349.92	375.17	401.31	439.55
	KT	Rejected	Rejected	Rejected	Rejected	Rejected
$10^{-6}$	α	1.963816	1.933809	1.933809	1.933809	1.933809
	β	0.984264	0.030693	0.030699	0.030694	0.030695
	μ	0.000005	0.000001	0.000001	0.000001	0.000001
	σ	0.000531	0.000518	0.000518	0.000518	0.000518
	PT	326.91	394.04	429.73	457.26	504.21
	KT	Rejected	Accepted	Accepted	Accepted	Accepted
$10^{-7}$	α	1.969975	1.933809	1.933809	1.933809	1.933809
	β	0.981810	0.030695	0.030696	0.030695	0.030695
	μ	−0.000000	0.000001	0.000001	0.000001	0.000001
	σ	0.000528	0.000518	0.000518	0.000518	0.000518
	PT	359.80	398.40	432.18	469.62	511.38
	KT	Rejected	Accepted	Accepted	Accepted	Accepted
$10^{-8}$	α	1.969975	1.933809	1.933809	1.933809	1.933809
	β	0.981810	0.030695	0.030696	0.030695	0.030695
	μ	−0.000000	0.000001	0.000001	0.000001	0.000001
	σ	0.000528	0.000518	0.000518	0.000518	0.000518
	PT	362.98	401.10	436.79	466.31	515.46
	KT	Rejected	Accepted	Accepted	Accepted	Accepted

**Table 4:** Dependence of 29 DAX returns series processing time (sec) with 64 processes (for time step Δt=10) on the accuracy of the probability density function (4) calculation ($\varepsilon_{pdf}$) and the accuracy of the maximum likelihood optimization ($\varepsilon_{ML}$). The number of stagnated series for every accuracy pair ($\varepsilon_{pdf}$ and $\varepsilon_{ML}$) is given in parentheses.

	$\varepsilon_{\mathrm{pdf}}$				
$\varepsilon_{\mathrm{ML}}$	$10^{-8}$	$10^{-9}$	$10^{-10}$	$10^{-11}$	$10^{-12}$
$10^{-5}$	3400.24 (-)	3934.80 (-)	4358.86 (-)	4898.37 (-)	5604.82 (-)
$10^{-6}$	3763.15 (-)	4269.03 (-)	4727.41 (-)	5242.13 (-)	6062.17 (-)
$10^{-7}$	8671.90 (3)	5762.27 (1)	6224.88 (1)	6088.49 (2)	6403.56 (-)
$10^{-8}$	8810.85 (3)	9238.92 (3)	8484.65 (3)	6268.37 (3)	12,094.20 (3)

**Table 5:** Maximum-likelihood estimates of mixed-stable parameters for 29 DAX returns series with time step Δt=10. The accuracy of the calculation of the probability density function $\varepsilon_{pdf}=10^{-12}$ and the accuracy of the maximum likelihood optimization $\varepsilon_{ML}=10^{-7}$. KT stands for the outcome of the Koutrouvelis test (0, “rejected”; 1, “not rejected”).

Company	*r*	α	β	μ	σ	KT
Adidas AG	0.75	1.813224	0.004395	0.000002	0.000456	1
Deutsche Bank	0.47	1.822488	−0.013724	−0.000001	0.000272	1
BASF SE	0.58	1.798121	0.009887	0.000001	0.000292	1
BMW AG St	0.68	1.872899	−0.024771	0.000000	0.000405	1
Continental AG	0.66	1.704703	−0.007413	0.000000	0.000363	1
Deutsche Post	0.75	1.933809	0.030695	0.000001	0.000518	1
Deutsche Telekom	0.73	1.995341	−0.064854	0.000001	0.000572	0
Bayer AG O.N.	0.60	1.875897	0.023037	0.000001	0.000364	1
FMC AG	0.77	1.759640	−0.014327	0.000000	0.000484	0
Deutsche Börse	0.68	1.664209	0.015807	0.000003	0.000413	1
MAN SE St	0.68	1.669219	0.001631	0.000002	0.000443	1
Henkel AG	0.77	1.769518	−0.031952	0.000000	0.000511	0
Infineon Techn.	0.82	1.979982	−0.045985	−0.000004	0.000803	0
Linde AG	0.74	1.714015	−0.007574	0.000002	0.000367	1
Merck KGaA	0.77	1.612534	0.003719	0.000000	0.000442	1
RWE AG St	0.58	1.852625	0.025781	0.000001	0.000330	1
Daimler AG	0.49	1.853430	0.039817	0.000001	0.000322	1
SAP AG	0.58	1.919404	0.012302	0.000000	0.000384	1
Siemens AG	0.46	1.815861	−0.002928	0.000001	0.000276	1
METRO AG St	0.75	1.767016	0.044610	0.000004	0.000393	1
ThyssenKrupp	0.68	1.855014	−0.000734	0.000001	0.000461	1
Volkswagen AG St	0.59	1.744243	−0.004622	0.000002	0.000302	1
Deutsche Postbank	0.79	1.678412	−0.005400	0.000000	0.000519	1
HYPO RE	0.75	1.814879	0.027742	0.000000	0.000576	1
Commerzbank AG	0.66	1.901063	−0.007980	0.000000	0.000477	1
Deutsche Lufthansa	0.78	1.932395	−0.016547	0.000000	0.000587	1
Allianz SE	0.43	1.787790	−0.016081	0.000000	0.000255	1
Münchener Rück	0.59	1.779122	0.006533	0.000000	0.000271	1
TUI AG	0.80	1.903315	0.012532	0.000003	0.000699	1

## Data Availability

The data used in this work are available from the corresponding author upon reasonable request.

## Abbreviations

The following abbreviations are used in this manuscript:

DAX	Deutscher Aktienindex
PT	Processing time
KT	Koutrouvelis test
ML	Maximum likelihood
pdf	probability density function

## References

[1] Bailey G., Steeley J. (2019). Forecasting the volatility of the Australian dollar using high-frequency data: Does estimator accuracy improve forecast evaluation?. Int. J. Financ. Econ..

[2] Degiannakis S., Filis G. (2018). Forecasting oil prices: High-frequency financial data are indeed useful. Energy Econ..

[3] Zhang Y., Wang J. (2019). Do high-frequency stock market data help forecast crude oil prices? Evidence from the MIDAS models. Energy Econ..

[4] Göncü A., Yang H. (2016). Variance-gamma and normal-inverse gaussian models: Goodness-of-fit to Chinese high-frequency index returns. N. Am. J. Econ. Financ..

[5] Belovas I. (2016). Modeling financial data distributions: A comparison of models. Computer Data Analysis and Modeling: Theoretical and Applied Stochastics. Proceedings of the XI International Conference.

[6] Koopman S., Lit R., Lucas A., Opschoor A. (2018). Dynamic discrete copula models for high-frequency stock price changes. J. Appl. Econom..

[7] Schabek T., Drazenovic B., Mance D. (2019). Reaction of Zagreb Stock Exchange CROBEX Index to macroeconomic announcements within a high frequency time interval. Zbornik Radova Ekonomskog Fakulteta u Rijeci časopis za Ekonomsku Teoriju i Praksu.

[8] Cai T.T., Hu J., Li Y., Zheng X. (2020). High-dimensional minimum variance portfolio estimation based on high-frequency data. J. Econom..

[9] Huang W., Gao X. (2021). LASSO-based high-frequency return predictors for profitable Bitcoin investment. Appl. Econ. Lett..

[10] Ambros M., Frenkel M., Huynh T.L.D., Kilinc M. (2020). COVID-19 pandemic news and stock market reaction during the onset of the crisis: Evidence from high-frequency data. Appl. Econ. Lett..

[11] Yousaf I., Ali S. (2020). The COVID-19 outbreak and high frequency information transmission between major cryptocurrencies: Evidence from the VAR-DCC-GARCH approach. Borsa Istanbul Rev..

[12] Kaklauskas L. (2012). Study and Application of Methods of Fractal Processes Monitoring in Computer Networks. Ph.D. Thesis.

[13] Cartea A., Jaimungal S., Penalva J. (2015). Algorithmic and High-Frequency Trading.

[14] Belovas I., Starikovičius V. (2015). Parallel computing for mixed-stable modelling of large data sets. Inf. Technol. Control.

[15] Belovas I., Sakalauskas L., Starikovičius V. (2017). A mixed-stable approach to the management of the portfolio using high-frequency financial data. Inf. Technol. Control.

[16] Belovas I., Starikovičius V. (2007). Parallelization of *α*-stable modelling algorithms. Math. Model. Anal..

[17] Belovas I., Kabašinskas A., Sakalauskas L. Returns modelling problem in the Baltic equity market. Proceedings of the Simulation and Optimisation in Business and Industry: International Conference on Operational Research.

[18] Kabašinskas A., Sakalauskas L., Sun W., Belovas I. (2012). Mixed-stable models for analyzing high-frequency financial data. J. Comput. Anal. Appl..

[19] Dacorogna R., Gençay U., Müller A., Olsen R., Pictet O. (2001). An Introduction of High-Frequency Finance.

[20] Nolan J.P. (2020). Univariate Stable Distributions. Models for Heavy Tailed Data.

[21] Cizek P., Hardle W., Weron R. (2011). Statistical Tools for Finance and Insurance.

[22] Cont R. (2001). Empirical properties of asset returns: Stylized facts and statistical issues. Quant. Financ..

[23] Celik N., Erden S., Sarikaya M. (2016). Comparing the estimation methods of stable distributions with respect to robustness properties. International Conference on Advances in Natural and Applied Sciences Proceedings.

[24] Corless R., Gonnet G., Hare D., Jeffrey D., Knuth D. (1996). On the Lambert W function. Adv. Comput. Math..

[25] Chapeau-Blondeau F., Monir A. (2002). Evaluation of the Lambert W Function and Application to Generation of Generalized Gaussian Noise With Exponent 1/2. IEEE Trans. Signal Process..

[26] Koutrouvelis I., Kellermeier J. (1981). Goodness-of-fit test based on the empirical characteristic function when parameters must be estimated. J. R. Stat. Soc. Ser. B.

